# A multi‐biomarker follow‐up study of patients with multiple sclerosis

**DOI:** 10.1002/brb3.509

**Published:** 2016-07-11

**Authors:** Morten Stilund, Mikkel Carstensen Gjelstrup, Tove Christensen, Holger Jon Møller, Thor Petersen

**Affiliations:** ^1^ Department of NeurologyAarhus University HospitalNørrebrogade 44DK‐8000Aarhus CDenmark; ^2^ Department of BiomedicineAarhus UniversityBartholin Building, Wilhelm Meyers Allé 4DK‐8000Aarhus CDenmark; ^3^ Department of Clinical BiochemistryAarhus University HospitalNørrebrogade 44DK‐8000Aarhus CDenmark

**Keywords:** Biomarker, cerebrospinal fluid, disease activity, multiple sclerosis, soluble CD163

## Abstract

**Objectives:**

This study aimed to examine the levels of the macrophage marker sCD163 and other biomarkers at the time of diagnosis of patients with either clinically isolated syndrome (CIS) or relapsing‐remitting multiple sclerosis (RRMS), and assess relation to clinical indicators of prognosis, disease activity (DA), and changes in the levels of these biomarkers at follow‐up.

**Materials and Methods:**

The clinical status and MRI were reevaluated in 56 patients more than 1 year after diagnosis with a median follow‐up time of 2 years. Levels of biomarkers in serum and cerebrospinal fluid (CSF) samples were evaluated by enzyme‐linked immunosorbent assays.

**Results:**

There was no significant difference in time to DA between patients with CIS and RRMS. A high sCD163 ratio (>0.07) was significantly (*P* = 0.04) associated with time to DA in the untreated patient group. In 21 patients reevaluated with serum and CSF samples, the sCD163 ratio levels decreased from 0.068 to 0.054 (*P* = 0.026) in the CIS/RRMS‐treated group. The CSF CXCL13, CXCL13 ratio, CSF neurofilament light polypeptide and osteopontin levels also decreased significantly in the CIS/RRMS‐treated group.

**Conclusions:**

The levels of all biomarkers changed concurrently with MS treatment. The sCD163 ratio was identified as a potential novel marker for time to DA.

## Introduction

Multiple sclerosis (MS) is generally assumed to be an autoimmune disease affecting the central nervous system (CNS) with inflammation and neurodegeneration as key elements in MS pathology (Frischer et al. 2009). Macrophages (MΦ) are abundantly present in the active and chronic lesions (plaques) of MS (Lucchinetti et al. 2000) and we have previously investigated the levels of the monocyte/MΦ‐specific protein, soluble CD163 (sCD163) at the time of diagnosis of MS (Stilund et al. 2014). Recently, we published an analysis of the diagnostic properties of sCD163 in relation to other biomarkers of inflammation and neurodegeneration in MS (Stilund et al. 2015). This study focuses on this specific combination of biomarkers for both neurodegeneration and inflammation, not only as diagnostic markers but also as novel markers potentially substantiating the prediction of prognosis and the monitoring of disease activity (DA) in MS.

Several factors such as glucocorticoids (Yeager et al. 2008), IL‐10, and IL‐6 upregulate the expression of CD163 on monocytes/MΦ, while IFN‐*γ*, TNF‐*α*, and GM‐CSF (granulocyte‐macrophage colony‐stimulating‐factor) promotes downregulation (Buechler et al. 2000). The CD163 expression is increased in MS plaques (Zhang et al. 2011). The sCD163 is a consequence of ectodomain shedding from the MΦ induced by inflammatory stimuli (Moller 2012), and increased sCD163 levels have been shown in patients with different stages of MS (Fabriek et al. 2007; Stilund et al. 2014, 2015). Although sCD163 has been shown to be a prognostic marker in a number of inflammatory diseases, such as rheumatoid arthritis, diabetes, and scleroderma, this has hitherto not been investigated in MS (Moller 2012).

There is a great demand for prognostic and predictive biomarkers in MS due to the generally unpredictable course of the disease (Compston and Coles 2008). Some clinical markers are now well established as predictors of disease prognosis (Confavreux et al. 2003); however, none of these clinical markers allows the clinician to perform an accurate short‐term prognosis. There is also no consensus on the prognostic value of neither routine cerebrospinal fluid (CSF) biomarkers (total protein, IgG index, and oligoclonal bands; Mandrioli et al. 2008; Becker et al. 2015) nor experimental biomarkers, such as those we present in this study (Brettschneider et al. 2010; Gout et al. 2011; Khademi et al. 2011; Teunissen and Khalil 2012).

The biomarkers selected for the study are known for their relation to monocyte/MΦ activation: NEO and sCD163 are both known to be produced by MΦ during acute and chronic inflammatory conditions; OPN enhances phagocytosis and acts as a monocyte/MΦ chemotactic agent; CXCL13 is a chemokine and its ligand, CXCR5, is found on B cells, T cells, and also monocyte/MΦs. Finally, NfL is a marker of axonal damage and is phagocytized by monocyte/MΦs (Stilund et al. 2014, 2015). This study evaluates the biomarker baseline and follow‐up levels for: (1) their relation to time to DA, (2) their correlation with known clinical markers of prognosis, and/or clinical and MRI markers of DA, and (3) the changes in biomarker levels during treatment.

## Patients and Methods

### Ethics statement

The study was conducted in accordance with the Ethical Declaration of Helsinki and all patients gave written, informed consent. The study and the material for informed consent were approved by The Central Denmark Region Committee on Biomedical Research Ethics (journal number: 20090210).

### Patient cohort

**Figure 1:**
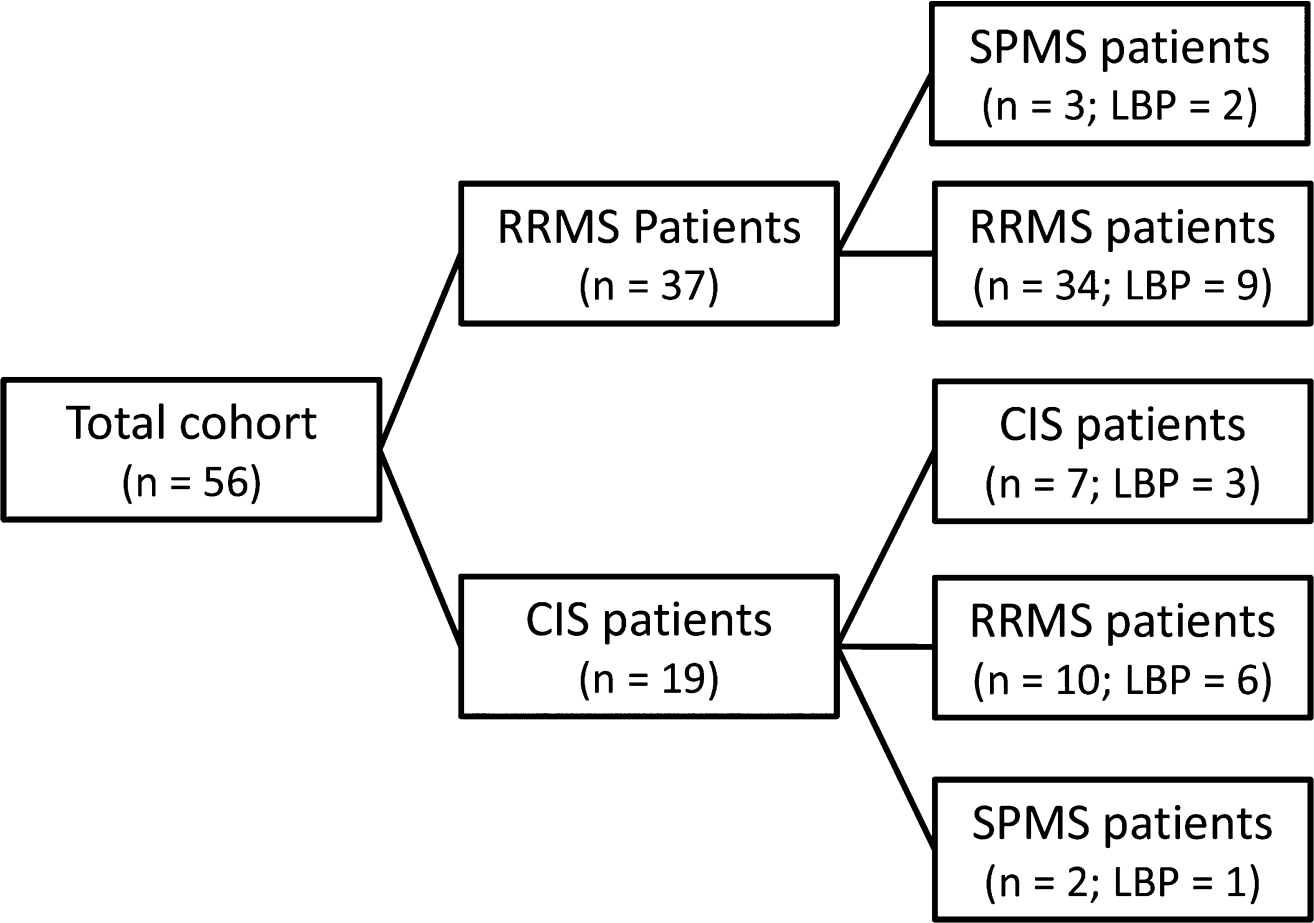
Flowchart presenting the follow‐up cohort. Patients included were followed up from our previous studies on diagnostic biomarkers (Stilund et al. 2014, 2015). This cohort comprises patients who volunteered to participate in a follow‐up clinical evaluation >1 year after diagnosis. Abbreviations: RRMS, relapsing‐remitting MS; CIS, clinically isolated syndrome; SPMS, secondary progressive MS;* n*, number of patients; LBP, lumbar puncture.

A total of 56 patients were included, 19 with clinically isolated syndrome (CIS) and 37 with relapsing‐remitting multiple sclerosis (RRMS). At follow‐up, 10 CIS patients had converted to RRMS and three of the initial RRMS patients had converted to secondary progressive MS (SPMS), as shown in the Cohort Flowchart (Fig. 1). All participants were rescanned with MRI of the neural axis and asked to participate in our biomarker study, contributing a lumbar puncture (LBP) and blood samples.

The follow‐up was conducted at the MS clinic, Department of Neurology, Aarhus University Hospital (AUH) and included medical history and anamnesis after diagnosis, and a clinical examination with an EDSS (extended disability status scale) score assessed according to Kurtzke (1983). Re‐LBP was performed in 21 patients with CSF analyses (cells, protein, and immunoglobulin G (IgG) index). None of the patients had received methylprednisolone or had a relapse at least 1 month prior to reevaluation.

In the patients without follow‐up LBP serum values of biomarkers were not examined because optimal biomarker performance required CSF measurement as shown in previous investigations (Stilund et al. 2014, 2015).

Treatment was defined as a period of at least 12 months of immune modulating therapy before resampling and follow‐up. DA was defined with three categories: progression on MRI (new lesions on MRI) and/or progression in EDSS (increment of ≥0.5 sustained for at least 6 months) and/or new attack within the follow‐up period. Known clinical prognostic indicators were defined as shown in Table 1.

**Table 1:** Indicators of disease activity, and prognosis

Attack DA	New attack in follow‐up period
MRI DA	New lesions or new Gd‐enhancing lesions at follow‐up
EDSS DA	Increase in EDSS of ≥0.5 at follow‐up (and lasting for at least 6 months after follow‐up)
Total DA	Combined score of Attack DA, MRI DA, and EDSS DA
Time to new attack	The time from baseline sampling to new attack^a^
MRI prognosis	Baseline MRI with ≥9 lesions is sign of poor prognosis
Debut symptom(s)	ON or sensory symptoms versus motor or cerebellar symptoms, the latter having a poorer prognosis
Attack‐rate	>1 attack in the first year before diagnosis is a sign of poor prognosis
Attack‐remission	Lack of attack‐remission at baseline is sign of poor prognosis
Age	Older age at time of diagnosis is sign of poor prognosis
Gender	Male patients have poorer prognosis

DA, disease activity; MRI, magnetic resonance imaging; EDSS, expanded disability status scale; Gd, gadolinium; ON, opticus neuritis.

The indicators used in the correlation analysis for disease activity and prognosis.

a Note that only nine patients in the resample group had a new attack in follow‐up period.

Secondary progressive MS was diagnosed with continuous progression of disability over more than 1 year without relapses. Lack of remission after the first relapse was defined as persistent deficit after more than 1 year. Demographics and paraclinical findings are summarized in Table 2.

**Table 2:** Demographic, clinical, and paraclinical data on follow‐up cohort patients with CIS or RRMS

Characteristics	CIS	RRMS
No. of Subjects, total *N* = 56 (follow‐up = 21)^a^	*N* = 19 (10)	*N* = 37 (11)
Gender M/F	5/14	5/32
Mean Age^b^ (range)	38 (18–74)	40 (25–65)
Mean EDSS at diagnosis (range)	2.0 (0–3.0)	2.5 (0–4.0)
Mean EDSS at follow‐up (range)	1.5 (0–5.0)	2.5 (0–6.5)
Mean No. of attacks at diagnosis^c^ (range)	1 (1–2)	3 (1–5)
Mean Follow‐up period in years (range)	2.0 (1.5–3.0)	2.0 (1.0–3.5)
Mean Disease Duration at follow‐up (range)^d^	38 (19–92)	79 (22–302)
Time since last attack at diagnosis (range)^e^	8 (0.1–48)	4 (3.3–14.5)
Time to first attack after diagnosis (range)^f^	11.6 (1.2–28.5)	9.5 (0.2–31.3)
*N* progressed/nonprogressed (percentage)	15/4 (81/19)	30/7 (79/21)
Mean baseline CSF Protein, *μ*mol/L (range)	0.44 (0.26–0.74)	0.39 (0.22–0.59)
Mean follow‐up CSF Protein, *μ*mol/L (range)	0.42 (0.26–0.66)	0.39 (0.22–0.56)
Mean baseline CSF Cells, 10^6^/L (range)	3.0 (1–55)	9.6 (0–40)
Mean follow‐up CSF Cells, 10^6^/L (range)	3.5 (0–8)	3.6 (1–13)
Mean baseline IgG Index (range)	1.01 (0.42–2.81)	1.17 (0.44–3.04)
Mean follow‐up IgG Index (range)	0.83 (0.41–1.59)	0.89 (0.46–1.65)
Mean total number of MRI white matter lesions (range)	9 (0–42)	18 (4–55)
Number of TR/UT at follow‐up	11/8	27/10
Pt resampled TR/UT	10 (5/5)	11 (8/3)

CIS, clinically isolated syndrome; RRMS, relapsing‐remitting MS; *N*, number of persons; EDSS, Expanded Disability Status Scale; N/A, not applicable or available; CSF, cerebrospinal fluid; TR, treated; UT, untreated.

a Refers to the patients included at baseline (*N* = 56) and patients who agreed to have resample of serum and CSF (*N* = 21).

b Age (in years).

c Mean number of attacks: mean number of attacks before the baseline sampling time point.

d Disease duration (in months): the period of time from debut of first symptom(s) to the baseline sampling time point.

e Time (in months) since last attack: the period of time from latest attack to the baseline sampling time point.

f Time (in months) to first attack after baseline lumbar puncture.

### ELISA analyses

Levels of all biomarkers were analyzed by enzyme‐linked immunosorbent assays (ELISA), essentially as previously described (Stilund et al. 2015) following the instructions given by the manufacturers. The concentration of sCD163 was analyzed in duplicate using an in‐house sandwich ELISA, essentially as described previously (Moller et al. 2002). Levels of CXCL13, NEO, and OPN were analyzed in both serum and CSF, and levels of NfL were only analyzed in CSF since the available kits for NfL, at the time of analysis, were restricted to be used only with CSF. Samples were run in duplicates and a coefficient of variation was calculated for each sample, accepting only values ≤15%. Intra‐assay variations were also calculated from six individual measurements of a known standard divided on each plate and values ≤15% were accepted (ranges: CXCL13 [2.02–8.57], NEO [3.54–13.67], NF‐Light [0.58–5.94%], and OPN [2.00–9.80%]). Samples with values exceeding the highest point of the standard curve were reanalyzed on a diluted sample. The diagnoses were established before the results of the sCD163 analyses were received and for the other four biomarkers, each plate contained 36 randomly selected samples marked with a study ID and assayed in a manner, blinded to the clinical status of the patients.

All ELISA plates were read on an ELISA plate reader (Multiscan FC; Thermo Scientific, Waltham, MA) at 450 and 540 or 620 nm, and concentrations were calculated by four parametric statistical regression in GraphPad Prism or linear regression in MS Excel. Handling of samples and ELISA analyses were performed as described previously (Stilund et al. 2015).

### Collection of data and statistical analysis

Data were stored and handled according to the Danish law on personal data. During collection of demographic data, we used the Electronic Patient Journal. Descriptions of MRI data were conducted by a neuroradiologist and confirmed by a senior neurologist who viewed all scans in the IMPAX system at the Department of Neurology, AUH.

For data collection, we used Microsoft Excel and all statistical analyses were performed using STATA12. See Table S1 for basic data.

We used right censored data in a time to event analysis with Kaplan–Meier estimates to evaluate DA and nonparametric log‐rank test on all biomarkers as DA predictors in treated (TR) and untreated (UT) patients.

## Results

The 56 patients were followed at the MS clinic for a minimum of 1 year and up to 3.5 years, median follow‐up time was 2 years (Table 1).

**Figure 2:**
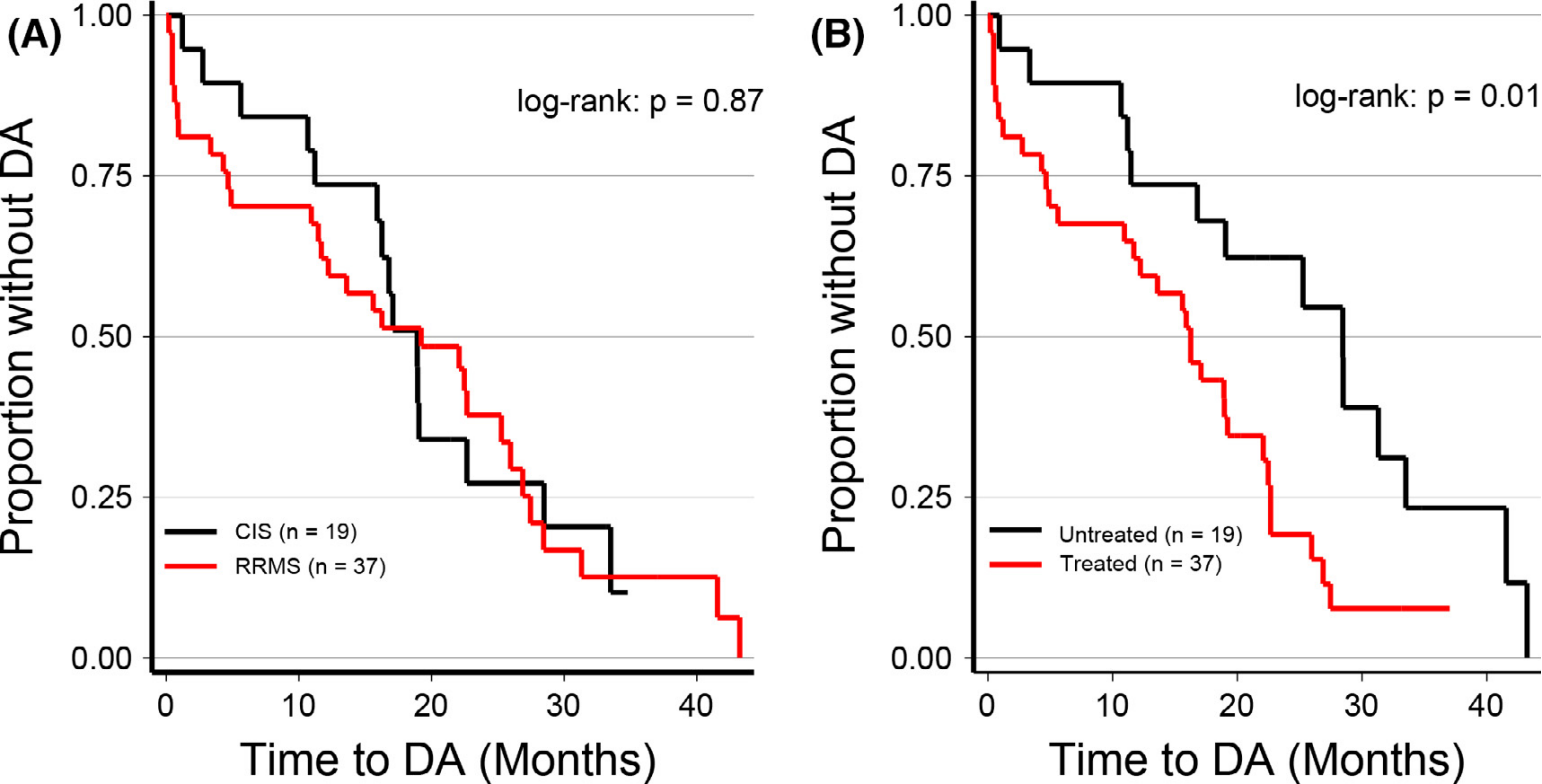
The Kaplan–Meier DA estimates in patients with baseline CIS and RRMS (A) and in treated and untreated patients (B). The plot shows the proportion of patients without DA (no attack, no EDSS, or MRI progression) as a function of time to DA (Months). We performed the nonparametric log‐rank test for statistics. Abbreviations: CIS, clinically isolated syndrome; RRMS, relapsing‐remitting MS; DA, disease activity.

The number of patients registered with increased clinical and MRI markers of DA was 47 (27 with new relapse, 24 with new MRI lesion, and/or 20 with progression on EDSS). Although patients with CIS showed less progression during the first 12 months compared to patients with RRMS (Fig. 2A), there was no significant difference in time to DA over the entire study period (*P* = 0.87). The mean follow‐up periods of the two patient groups were equal. As shown in Figure 2B, time to DA was significantly shorter in the treated group compared to the nontreated group (*P* < 0.01).

A significantly higher number of patients with CIS (as compared to RRMS) progressed on MRI (*P* = 0.01). In the treated group, a significantly higher number of new MRI lesions was found (*P* = 0.02), but there were no significant differences between the groups with regards to time to EDDS progression or time to new attack (see Tables S7 and S8).

### Correlation of baseline clinical markers of prognosis, and/or clinical and MRI markers of DA

Several of the biomarkers were significantly correlated (see Tables S2–S21) to baseline clinical markers of prognosis, and/or clinical and MRI markers of DA (presented in Table 1) in univariate analyses.

The CSF sCD163 baseline level was correlated with MRI DA (*r* = −0.28; *P* = 0.04), EDSS DA (*r* = −0.33; *P* = 0.01), and Total DA (*r* = −0.33; *P* = 0.02). The serum sCD163 levels were correlated with MRI prognosis (*r* = 0.27, *P* = 0.04). The sCD163 ratio did not show any correlation with clinical markers of prognosis, and/or clinical and MRI markers of DA. There was no correlation between CXCL13 and clinical and MRI markers of DA, but the prognostic indicator: attack‐remission, was correlated with the CXCL13 CSF (*r* = 0.33; *P* = 0.01) and the CXCL13 ratio (*r* = 0.28; *P* = 0.03). Age was correlated with the CXCL13 CSF (*r* = −0.40; *P* < 0.01), the CXCL13 serum (*r* = −0.42; *P* < 0.01), and the CXCL13 ratio (*r* = −0.30; *P* = 0.02). NEO was not correlated with baseline clinical markers of prognosis, and/or clinical and MRI markers of DA. The OPN CSF correlated with time from sampling to new attack (*r* = 0.61; *P* < 0.01) and the NfL CSF correlated with attack‐remission (*r* = 0.29; *P* = 0.03). The clinical prognostic indicators did not show significant relation to DA in this study.

With Bonferroni correction, none of the biomarkers were significantly correlated with any of the clinical indicators of DA or any of the clinical, demographic, and MRI indicators of prognosis.

### Survival time analysis of biomarker levels and time to DA

**Figure 3:**
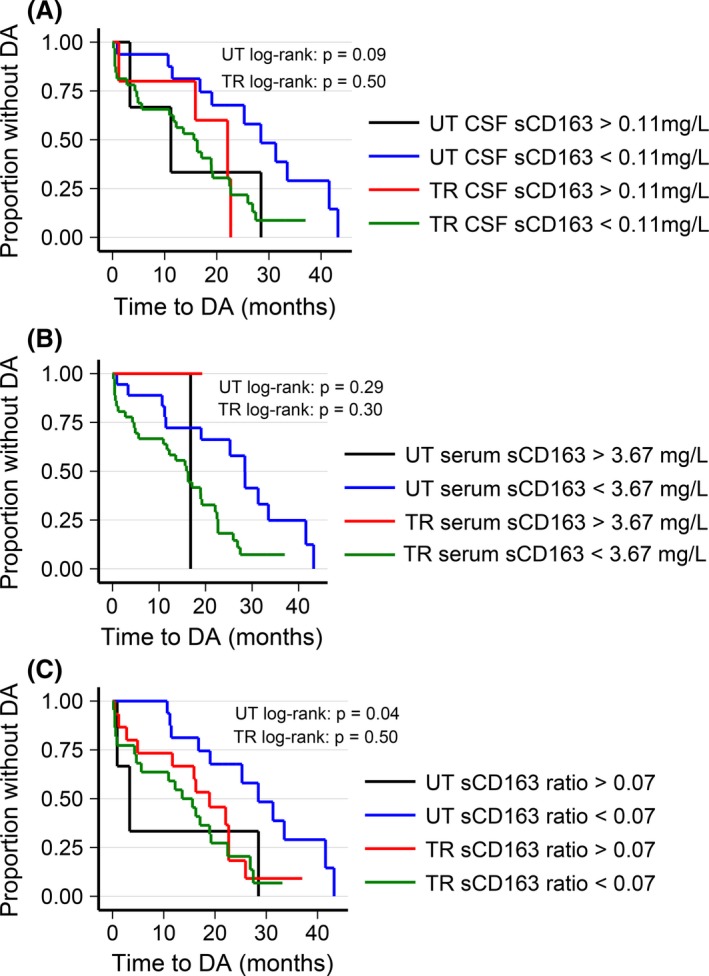
The untreated and treated patients and their DA rates in relation to levels of the bio marker sCD163. (A–C) are Kaplan–Meier curves illustrating time to DA from diagnosis in UT and TR patients with CIS/RRMS based on CSF sCD163 (A), serum sCD163 (B), and sCD163 ratio (C). Log‐rank test for equality of survivor functions were performed on UT and TR patients with CIS/RRMS, respectively. Abbreviations: UT, untreated; TR, treated; DA, disease activity; CIS, clinically isolated syndrome; RRMS, relapsing‐remitting MS.

The cut points for sCD163 are shown in Figure 3. Here, we also present the Kaplan–Meier plots and the corresponding nonparametric log‐rank tests, which were performed on untreated (UT) and treated (TR) patients with CIS/RRMS, respectively. A high sCD163 ratio was significantly associated to time to DA in the untreated patient group (log‐rank test: *P*‐value = 0.04) (Fig. 3). Other log‐rank tests on sCD163 were not significant. Similar Kaplan–Meier plots were produced for CXCL13, NfL, NEO, and OPN but log‐rank tests were not significant for any of these biomarkers.

### Biomarkers as indicators of response to treatment

**Figure 4:**
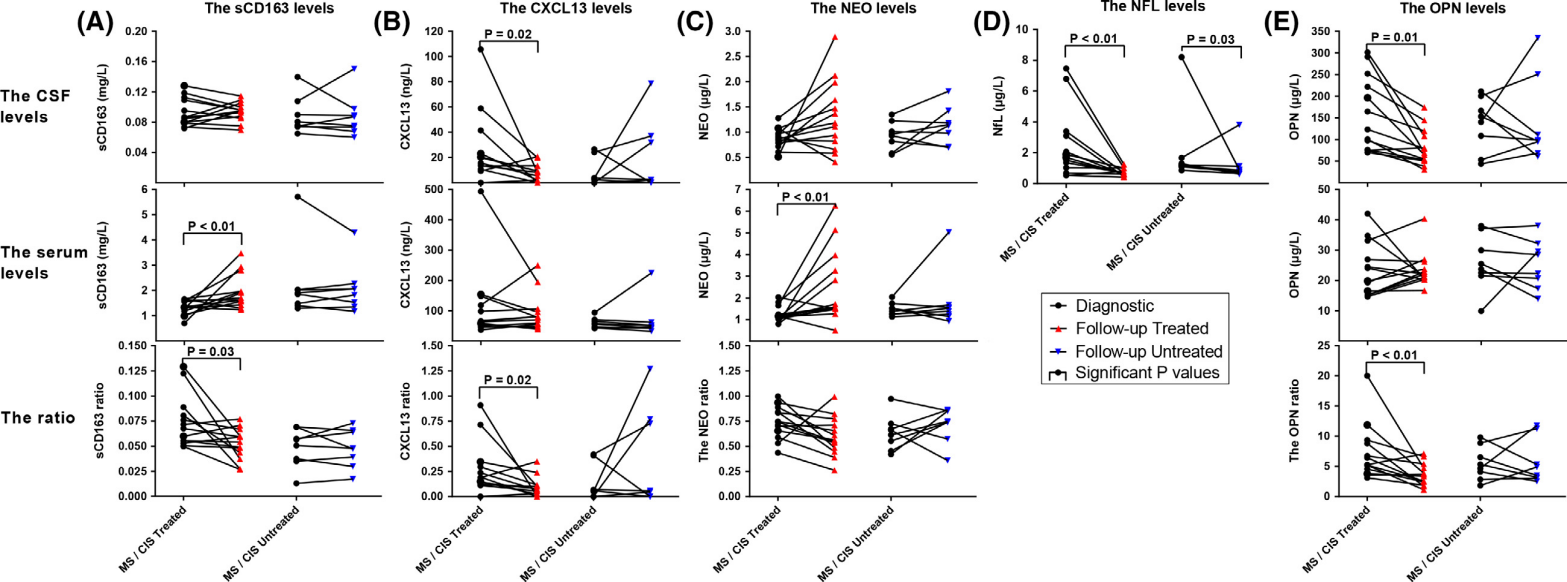
Biomarker levels in treated or untreated patients. Changes in the CSF and serum levels in the follow‐up cohort in treated and untreated patients are shown. In the far left column (A) we present the levels of CSF sCD163, serum sCD163, and ratio sCD163 for treated and untreated patients at the time of diagnosis and after a minimum of 1 year follow‐up time. In the columns (B–E) to the right, data are shown for CXCL13, NEO, NfL, and OPN. Each graph shows the biomarker levels in treated or untreated patients with CIS and RRMS. *U*‐tests (Mann–Whitney nonparametric test) of the difference between diagnostic and follow‐up levels are marked by a bar and corresponding *P*‐values. Abbreviations: CIS, clinically isolated syndrome; RRMS, relapsing‐remitting MS; NEO, neopterin; NfL, neurofilament light polypeptide; OPN, osteopontin.

Changes in the CSF and serum levels of the biomarkers during follow‐up are shown in Figure 4. In the first section of Figure 4, we present the levels of CSF sCD163, serum sCD163, and ratio sCD163 for 13 treated and eight untreated patients with CIS/RRMS at the time of diagnosis and after a minimum of 1 year follow‐up time. In the following sections in Figure 4, we present the data for CXCL13, NfL, NEO, and OPN, further described in detail in Supplementary Data Tables S22 and S23 and Figure S1.

Three patients had a marked reduction in levels of more than one of the biomarkers. The serum sCD163 levels increased significantly in the CIS/RRMS‐treated group from 1.336 to 1.706 mg/L (*P* = 0.04) and the levels of the corresponding sCD163 ratio decreased from 0.068 to 0.054 (*P* = 0.026). The CSF CXCL13 and the levels of the CXCL13 ratio decreased significantly in the CIS/RRMS‐treated group from 15.50 to 8.156 ng/L (*P* = 0.022) and 0.188 to 0.066 (*P* = 0.022), respectively. We also found a significant increase in the serum NEO levels in treated patients from 1.132 to 1.565 *μ*g/L (*P* = 0.007). There was a decrease in the levels of CSF NfL in both the TR and UT patients from baseline 1.698 to 0.672 *μ*g/L (*P* = 0.004) and 1.207 to 0.831 *μ*g/L (*P* = 0.028), respectively. Finally, the CSF OPN and OPN ratio were significantly decreased from 123.2 to 69.17 *μ*g/L (*P* = 0.010) and 5.276 to 3.394 (*P* = 0.009), respectively.

## Discussion

In this section, we will discuss the four main findings of the study: (1) the study cohort had progression rates that were similar to previous studies. (2) Our correlation analyses showed that several baseline biomarkers were related to baseline clinical markers of prognosis, and/or clinical and MRI markers of DA. This, however, was not the case when corrected with Bonferroni. (3) sCD163 ratio has potential as an indicator of time to DA in untreated patients. (4) The levels of all biomarkers changed concurrently with MS treatment.

The time to DA for patients with CIS was similar to previous reports (D'Alessandro et al. 2013), showing a median time for conversion into definite MS of approximately 2 years. Also, regarding progression on MRI, similar results have been reported, with new MRI activity in approximately 50% of patients within a 2‐year follow‐up period (Freedman 2012). The patients with CIS and RRMS (Lublin et al. 2014) were classified upon follow‐up as treated or untreated with the untreated group representing a benign course of the disease (Pittock et al. 2004; Glad et al. 2011). The rationale for not starting treatment was based on the well‐known prognostic clinical findings such as: only slight disturbing initial symptoms, complete remission of the relapse and afterward no daily symptoms. Two patients were not started on treatment since they converted to the secondary progressive phase of the disease.

In the correlation analyses, we examined whether baseline clinical markers of prognosis, and/or clinical and MRI markers of DA were related to baseline biomarker levels. Without Bonferroni, some of these biomarkers were significantly correlated and should be validated in a larger prospective cohort. Insignificant correlation of biomarkers in relation to clinical and MRI markers of DA could be explained by the effect of treatment. We therefore analyzed treated and untreated patients dichotomized by cut points of biomarker levels in a time to event analysis. This analysis identified sCD163 ratio as a potential indicator of time to DA in untreated patients. None of the other biomarkers showed this relation in the time to event analysis; however, the biomarker cut points should also be validated in a cohort of healthy controls.

The main finding in this study was that biomarker levels did change with treatment when comparing diagnostic sample levels with follow‐up sample levels. One of the caveats for this result is whether the effect on biomarker levels is caused by treatment or whether it is fluctuations representing the natural course of the disease. The CSF NfL levels also differed between untreated baseline and follow‐up level; however, the cause for this difference is the presence of one extreme outlier in the CSF NfL untreated baseline group and when removed, this level of significance was no longer present. The levels for all the other biomarkers in this group of untreated patients exhibited insignificant changes (Fig. 3). Additionally, the decrease in biomarkers levels in relation to treatment and DA demonstrated in this study, is substantiated by previous reports (Khademi et al. 2011; Holmoy et al. 2013; Kuhle et al. 2013; Sellebjerg et al. 2014).

In this study, 24 of the follow‐up patients received first‐line interferon beta (IFN‐*β*) therapy. Several of the proposed effects of IFN‐*β* were recently reviewed (Gonzalez‐Navajas et al. 2012) and involves monocytes, MΦ, and microglia. Further, a study from 2006 (Teige et al. 2006) showed that IFN‐*β* lead to a decrease in microglial cells’ ability to present antigens.

Recent studies have reported elevated levels of sCD163 in serum in a number of auto‐inflammatory diseases (Dige et al. 2014; Kazankov et al. 2015), whereas the baseline levels in this study were decreased in serum of patients with MS (Stilund et al. 2014). The reason for this difference is unknown; however, it may suggest that the recruitment of monocytes to the CNS has a depleting effect on the serum sCD163 levels in patients with MS. This finding is supported by the finding in our previous study that 90% of the sCD163 found in the CSF is produced intrathecally (Stilund et al. 2014). Upon treatment, the levels would be expected to increase, as was in fact the case.

As we have shown before (Stilund et al. 2015), NEO and sCD163 levels followed similar patterns (Fig. 3). Both biomarkers, which are markers of macrophage activity, exhibited significantly increased levels in serum at follow‐up in the treated group. The sCD163 ratio decreased significantly upon treatment as was also the case for OPN ratio and especially CXCL13 ratio, confirming previous findings (Sellebjerg et al. 2009). The decrease in the latter were due to a significant decrease in follow‐up CSF levels, possibly indicating a lesser recruitment of B cells (Carlsen et al. 2004) and monocytes (Sinclair et al. 2005) to the CNS compartment, and thus indicating decrease in DA. The decrease in inflammatory markers upon treatment has previously been shown for CXCL16 (Holmoy et al. 2013) and in the same study OPN levels, as an indicator of DA, were found unchanged as is also the case in this study. Finally CSF NfL, a marker of early axonal damage (Semra et al. 2002), has also in another study showed a significant decrease in treated patients at follow‐up, which could also be interpreted as an indication of lower DA (Kuhle et al. 2013).

Results from this study are thus, in agreement with other reports on biomarkers and their relation to DA in MS (O'Rourke et al. 2007; Sellebjerg et al. 2014). In our survival time analysis, the clinical assessment and biomarker evaluation showed a trend as indicators of risk of future DA which should be further developed and validated for accuracy in larger prospective cohorts of patients with MS and CIS. Interestingly, this study indicates that sCD163 levels are treatment dependent, and we suggest that sCD163 levels could be applied in future clinical trials as markers of DA and response to treatment.

## Conflict of Interest

The authors declare no financial or other conflicts of interest.

## Supporting information


**Table S1.** Basic data.
**Table S2.** Spearman correlation analysis with Bonferroni correction on the sCD163 biomarker levels, and clinical and MRI markers of disease activity.
**Table S3.** Spearman correlation analysis without Bonferroni correction on the sCD163 biomarker levels, and clinical and MRI markers of disease activity.
**Table S4.** Spearman correlation analysis with Bonferroni correction on the sCD163 biomarker levels, and baseline clinical markers of prognosis.
**Table S5.** Spearman correlation analysis without Bonferroni correction on the sCD163 biomarker levels, and baseline clinical, demographic, and MRI markers of prognosis.
**Table S6.** Spearman correlation analysis with Bonferroni correction on the CXCL13 biomarker levels, and clinical and MRI markers of disease activity.
**Table S7.** Spearman correlation analysis without Bonferroni correction on the CXCL13 biomarker levels, and clinical and MRI markers of disease activity.
**Table S8.** Spearman correlation analysis with Bonferroni correction on the CXCL13 biomarker levels, and baseline clinical markers of prognosis.
**Table S9.** Spearman correlation analysis without Bonferroni correction on the CXCL13 biomarker levels, and baseline clinical markers of prognosis.
**Table S10.** Spearman correlation analysis with Bonferroni correction on the NEO biomarker levels, and clinical and MRI markers of disease activity.
**Table S11.** Spearman correlation analysis without Bonferroni correction on the NEO biomarker levels, and clinical and MRI markers of disease activity.
**Table S12.** Spearman correlation analysis with Bonferroni correction on the NEO biomarker levels, and baseline clinical markers of prognosis.
**Table S13**. Spearman correlation analysis without Bonferroni correction on the NEO biomarker levels, and baseline clinical markers of prognosis.
**Table S14.** Spearman correlation analysis with Bonferroni correction on the NfL biomarker levels, and clinical and MRI markers of disease activity.
**Table S15.** Spearman correlation analysis without Bonferroni correction on the NfL biomarker levels, and clinical and MRI markers of disease activity.
**Table S16.** Spearman correlation analysis with Bonferroni correction on the NfL biomarker levels, and baseline clinical markers of prognosis.
**Table S17.** Spearman correlation analysis without Bonferroni correction on the NfL biomarker levels, and baseline clinical markers of prognosis.
**Table S18.** Spearman correlation analysis with Bonferroni correction on the OPN biomarker levels, and clinical and MRI markers of disease activity.
**Table S19.** Spearman correlation analysis without Bonferroni on the OPN biomarker levels, and clinical and MRI markers of disease activity.
**Table S20.** Spearman correlation analysis with Bonferroni correction on the OPN biomarker levels, and baseline clinical markers of prognosis.
**Table S21.** Spearman correlation analysis without Bonferroni correction on the OPN biomarker levels, and baseline clinical markers of prognosis.
**Table S22.** Biomarker levels at baseline and follow‐up in treated or untreated patients with CIS/RRMS.
**Table S23.** Biomarker levels in either treated or untreated patients with RRMS or CIS.
**Table S24.** Biomarker SD (standard deviation), and percentiles.
**Figure S1.** Biomarker levels in either treated or untreated patients with RRMS or CIS.
