# Supplementary material for: A multi‐biomarker follow‐up study of patients with multiple sclerosis
Source: Brain Behav. 2016 Jul 11;6(9):e00509. doi: 10.1002/brb3.509 (PMC5036432; doi:10.1002/brb3.509)
Supplement: Supplementary file 1 — Table S1. Basic data. Table S2. Spearman correlation analysis with Bonferroni correction on the sCD163 biomarker levels, and clinical and MRI markers of disease activity. Table S3. Spearman correlation analysis without Bonferroni correction on the sCD163 biomarker levels, and clinical and MRI markers of disease activity. Table S4. Spearman correlation analysis with Bonferroni correction on the sCD163 biomarker levels, and baseline clinical markers of prognosis. Table S5. Spearman correlation analysis without Bonferroni correction on the sCD163 biomarker levels, and baseline clinical, demographic, and MRI markers of prognosis. Table S6. Spearman correlation analysis with Bonferroni correction on the CXCL13 biomarker levels, and clinical and MRI markers of disease activity. Table S7. Spearman correlation analysis without Bonferroni correction on the CXCL13 biomarker levels, and clinical and MRI markers of disease activity. Table S8. Spearman correlation analysis with Bonferroni correction on the CXCL13 biomarker levels, and baseline clinical markers of prognosis. Table S9. Spearman correlation analysis without Bonferroni correction on the CXCL13 biomarker levels, and baseline clinical markers of prognosis. Table S10. Spearman correlation analysis with Bonferroni correction on the NEO biomarker levels, and clinical and MRI markers of disease activity. Table S11. Spearman correlation analysis without Bonferroni correction on the NEO biomarker levels, and clinical and MRI markers of disease activity. Table S12. Spearman correlation analysis with Bonferroni correction on the NEO biomarker levels, and baseline clinical markers of prognosis. Table S13. Spearman correlation analysis without Bonferroni correction on the NEO biomarker levels, and baseline clinical markers of prognosis. Table S14. Spearman correlation analysis with Bonferroni correction on the NfL biomarker levels, and clinical and MRI markers of disease activity. Table S15. Spearman correlation analysis withou [file BRB3-6-e00509-s001.docx]

# Supplementary Data

## **Index of tables and figures**

[Supplementary Data 1](#_Toc447564108)

[Index of tables and figures 1](#_Toc447564109)

[Table S1 Basic Data 3](#_Toc447564110)

[Statistical analysis 4](#_Toc447564111)

[Table S2 Spearman correlation analysis with Bonferroni correction on the sCD163 biomarker levels, and clinical and MRI markers of disease activity 5](#_Toc447564112)

[Table S3 Spearman correlation analysis without Bonferroni correction on the sCD163 biomarker levels, and clinical and MRI markers of disease activity 6](#_Toc447564113)

[Table S4 Spearman correlation analysis with Bonferroni correction on the sCD163 biomarker levels, and baseline clinical markers of prognosis 7](#_Toc447564114)

[Table S5 Spearman correlation analysis without Bonferroni correction on the sCD163 biomarker levels, and baseline clinical, demographic, and MRI markers of prognosis 8](#_Toc447564115)

[Table S6 Spearman correlation analysis with Bonferroni correction on the CXCL13 biomarker levels, and clinical and MRI markers of disease activity 9](#_Toc447564116)

[Table S7 Spearman correlation analysis without Bonferroni correction on the CXCL13 biomarker levels, and clinical and MRI markers of disease activity 10](#_Toc447564117)

[Table S8 Spearman correlation analysis with Bonferroni correction on the CXCL13 biomarker levels, and baseline clinical markers of prognosis 11](#_Toc447564118)

[Table S9 Spearman correlation analysis without Bonferroni correction on the CXCL13 biomarker levels, and baseline clinical markers of prognosis 12](#_Toc447564119)

[Table S10 Spearman correlation analysis with Bonferroni correction on the NEO biomarker levels, and clinical and MRI markers of disease activity 13](#_Toc447564120)

[Table S11 Spearman correlation analysis without Bonferroni correction on the NEO biomarker levels, and clinical and MRI markers of disease activity 14](#_Toc447564121)

[Table S12 Spearman correlation analysis with Bonferroni correction on the NEO biomarker levels, and baseline clinical markers of prognosis 15](#_Toc447564122)

[Table S13 Spearman correlation analysis without Bonferroni correction on the NEO biomarker levels, and baseline clinical markers of prognosis 16](#_Toc447564123)

[Table S14 Spearman correlation analysis with Bonferroni correction on the NfL biomarker levels, and clinical and MRI markers of disease activity 17](#_Toc447564124)

[Table S15 Spearman correlation analysis without Bonferroni correction on the NfL biomarker levels, and clinical and MRI markers of disease activity 18](#_Toc447564125)

[Table S16 Spearman correlation analysis with Bonferroni correction on the NfL biomarker levels, and baseline clinical markers of prognosis 19](#_Toc447564126)

[Table S17 Spearman correlation analysis without Bonferroni correction on the NfL biomarker levels, and baseline clinical markers of prognosis 20](#_Toc447564127)

[Table S18 Spearman correlation analysis with Bonferroni correction on the OPN biomarker levels, and clinical and MRI markers of disease activity 21](#_Toc447564128)

[Table S19 Spearman correlation analysis without Bonferroni on the OPN biomarker levels, and clinical and MRI markers of disease activity 22](#_Toc447564129)

[Table S20 Spearman correlation analysis with Bonferroni correction on the OPN biomarker levels, and baseline clinical markers of prognosis 23](#_Toc447564130)

[Table S21 Spearman correlation analysis without Bonferroni correction on the OPN biomarker levels, and baseline clinical markers of prognosis 24](#_Toc447564131)

[Table S22. Biomarker levels at baseline and follow-up in treated or untreated patients with CIS / RRMS 25](#_Toc447564132)

[Figure S1. Biomarker levels in either treated or untreated patients with RRMS or CIS 26](#_Toc447564133)

[Table S23. Biomarker levels in either treated or untreated patients with RRMS or CIS. 27](#_Toc447564134)

[Table S24: Biomarker SD (standard deviation), and percentiles 28](#_Toc447564135)

## Table S1 Basic Data

**Table S1 Basic data.** Excel file above (double click to activate) contains all basic data for this paper. Abbreviations: RRMS (relapsing-remitting MS), PPMS (primary-progressive MS), SPMS (secondary-progressive MS), CIS (clinically isolated syndrome), CSF (cerebrospinal fluid), y (years), Gender (1=male; 2=female), TNL (Total number of white matter lesions).

# Statistical analysis

We used right censored data in a time to event analysis with Kaplan-Meier estimates to evaluate DA and non-parametric log-rank test on all biomarkers as DA predictors in treated (TR) and untreated (UT) patients.

We performed a non-parametric Mann-Whitney U-test to evaluate the difference in biomarker levels at diagnosis and at follow-up in treated and untreated patients with either CIS or RRMS.

Spearman correlation analysis, with and without Bonferroni correction, was used to correlate variables that were on an ordinal scale. Since biomarkers in CSF and serum were positively skewed, we performed correlation analyses on LOG-transformed data. For calculations of ratios (e.g. the sCD163 ratio) the CSF concentration was divided by the serum concentration.

A survival time analysis was performed by using defined cut-points for dichotomization by calculation of 95 percentiles of the biomarkers from our previous established SC group (4). We did not use max or minimum levels of biomarkers as cut points in order to avoid exclusion by extreme outliers in the SC group (table S24 for details on these calculations). For a view on the data generated see Supplementary Data for STATA do-files and output.

## Table S2 Spearman correlation analysis with Bonferroni correction on the sCD163 biomarker levels, and clinical and MRI markers of disease activity

## Table S3 Spearman correlation analysis without Bonferroni correction on the sCD163 biomarker levels, and clinical and MRI markers of disease activity

## Table S4 Spearman correlation analysis with Bonferroni correction on the sCD163 biomarker levels, and baseline clinical markers of prognosis

## Table S5 Spearman correlation analysis without Bonferroni correction on the sCD163 biomarker levels, and baseline clinical, demographic, and MRI markers of prognosis

## Table S6 Spearman correlation analysis with Bonferroni correction on the CXCL13 biomarker levels, and clinical and MRI markers of disease activity

## Table S7 Spearman correlation analysis without Bonferroni correction on the CXCL13 biomarker levels, and clinical and MRI markers of disease activity

## Table S8 Spearman correlation analysis with Bonferroni correction on the CXCL13 biomarker levels, and baseline clinical markers of prognosis

## Table S9 Spearman correlation analysis without Bonferroni correction on the CXCL13 biomarker levels, and baseline clinical markers of prognosis

## Table S10 Spearman correlation analysis with Bonferroni correction on the NEO biomarker levels, and clinical and MRI markers of disease activity

## Table S11 Spearman correlation analysis without Bonferroni correction on the NEO biomarker levels, and clinical and MRI markers of disease activity

## Table S12 Spearman correlation analysis with Bonferroni correction on the NEO biomarker levels, and baseline clinical markers of prognosis

## Table S13 Spearman correlation analysis without Bonferroni correction on the NEO biomarker levels, and baseline clinical markers of prognosis

## Table S14 Spearman correlation analysis with Bonferroni correction on the NfL biomarker levels, and clinical and MRI markers of disease activity

## Table S15 Spearman correlation analysis without Bonferroni correction on the NfL biomarker levels, and clinical and MRI markers of disease activity

## Table S16 Spearman correlation analysis with Bonferroni correction on the NfL biomarker levels, and baseline clinical markers of prognosis

## Table S17 Spearman correlation analysis without Bonferroni correction on the NfL biomarker levels, and baseline clinical markers of prognosis

## Table S18 Spearman correlation analysis with Bonferroni correction on the OPN biomarker levels, and clinical and MRI markers of disease activity

## Table S19 Spearman correlation analysis without Bonferroni on the OPN biomarker levels, and clinical and MRI markers of disease activity

##

## Table S20 Spearman correlation analysis with Bonferroni correction on the OPN biomarker levels, and baseline clinical markers of prognosis

## Table S21 Spearman correlation analysis without Bonferroni correction on the OPN biomarker levels, and baseline clinical markers of prognosis

| Table S22. Biomarker levels at baseline and follow-up in treated or untreated patients with CIS / RRMS | | | | | | |
| --- | --- | --- | --- | --- | --- | --- |
|  | **Treated** | | | **Untreated** | | |
| **Characteristics** | **Median diagnostic levels** | **Median**  **follow-up levels** | **U-test** | **Median diagnostic levels** | **Median**  **follow-up levels** | **U-test** |
| **CSF sCD163 (mg/L)** | **0.086** | **0.095** | **0.504** | **0.078** | **0.081** | **0.777** |
| **range** | **(0.072 - 0.128)** | **(0.070 - 0.115)** |  | **(0.065 - 0.140)** | **(0.060 - 0.150)** |  |
| **Serum sCD163 (mg/L)** | **1.336** | **1.706** | **0.004*** | **1.882** | **1.942** | **0.777** |
| **range** | **(0.703 - 1.673)** | **(1.238 - 3.476)** |  | **(1.298 - 5.712)** | **(1.179 - 4.291)** |  |
| **sCD163 ratio** | **0.068** | **0.054** | **0.026*** | **0.054** | **0.048** | **0.932** |
| **range** | **(0.050 - 0.129)** | **(0.027 - 0.077)** |  | **(0.013 - 0.069)** | **(0.017 - 0.073)** |  |
| **CSF CXCL13 (ng/L)** | **15.50** | **8.156** | **0.022*** | **3.029** | **2.296** | **0.821** |
| **range** | **(0.001 - 105.8)** | **(0.001 - 20.62)** |  | **(0.001 - 26.55)** | **(0.001 - 78.78)** |  |
| **Serum CXCL13 (ng/L)** | **65.43** | **77.89** | **0.946** | **56.82** | **48.69** | **0.323** |
| **range** | **(36.91 - 493.9)** | **(38.81 - 249.6)** |  | **(41.79 - 93.92)** | **(32.30 - 224.9)** |  |
| **CXCL13 ratio** | **0.188** | **0.066** | **0.022*** | **0.054** | **0.052** | **0.702** |
| **Range** | **(<0.000 - 0.909)** | **(<0.000 - 0.349)** |  | **(<0.000 - 0.421)** | **(<0.000 - 1.269)** |  |
| **CSF NEO (µg/L)** | **0.869** | **1.185** | **0.090** | **0.948** | **1.141** | **0.275** |
| **range** | **(0.514 - 1.279)** | **(0.408 - 2.891)** |  | **(0.558 - 1.351)** | **(0.695 - 1.813)** |  |
| **Serum NEO (µg/L)** | **1.132** | **1.565** | **0.007*** | **1.447** | **1.456** | **0.932** |
| **range** | **(0.786 - 2.034)** | **(0.496 - 6.257)** |  | **(1.130 - 2.043)** | **(0.930 - 5.040)** |  |
| **NEO ratio** | **0.741** | **0.563** | **0.064** | **0.637** | **0.744** | **0.193** |
| **range** | **(0.435 - 0.996)** | **(0.262 - 0.993)** |  | **(0.420 - 0.972)** | **(0.360 - 0.861)** |  |
| **CSF NfL (µg/L)** | **1.698** | **0.672** | **0.004*** | **1.207** | **0.831** | **0.028*** |
| **range** | **(0.524 - 7.472)** | **(0.415 - 1.242)** |  | **(0.866- 8.209)** | **(0.613 - 3.813)** |  |
| **CSF OPN (µg/L)** | **123.2** | **69.17** | **0.010*** | **146.7** | **97.42** | **0.777** |
| **range** | **(69.81- 301.6)** | **(30.31 -174.3 )** |  | **(43.66 - 211.2)** | **(60.73 - 335.0)** |  |
| **Serum OPN (µg/L)** | **19.82** | **22.12** | **0.504** | **24.58** | **25.36** | **0.777** |
| **range** | **(14.64 - 42.04)** | **(16.63 - 40.37)** |  | **(9.901 - 38.00)** | **(13.95 - 38.09)** |  |
| **OPN ratio** | **5.276** | **3.394** | **0.009*** | **5.017** | **4.204** | **0.932** |
| **range** | **(3.110 - 20.03)** | **(1.126 - 7.088)** |  | **(1.843 - 9.821)** | **(2.526 - 11.77)** |  |

**Table 22 Biomarker levels at baseline and follow-up in treated or untreated patients with CIS / RRMS.** Abbreviations: CIS (clinically isolated syndrome), RRMS (relapsing remitting MS), NEO (neopterin), NfL (neurofilament light polypeptide), OPN (osteopontin). U-test (Mann-Whitney non-parametric test) of difference between diagnostic and follow-up levels. Significant difference p<0.05 is marked by *).

## Figure S1. Biomarker levels in either treated or untreated patients with RRMS or CIS


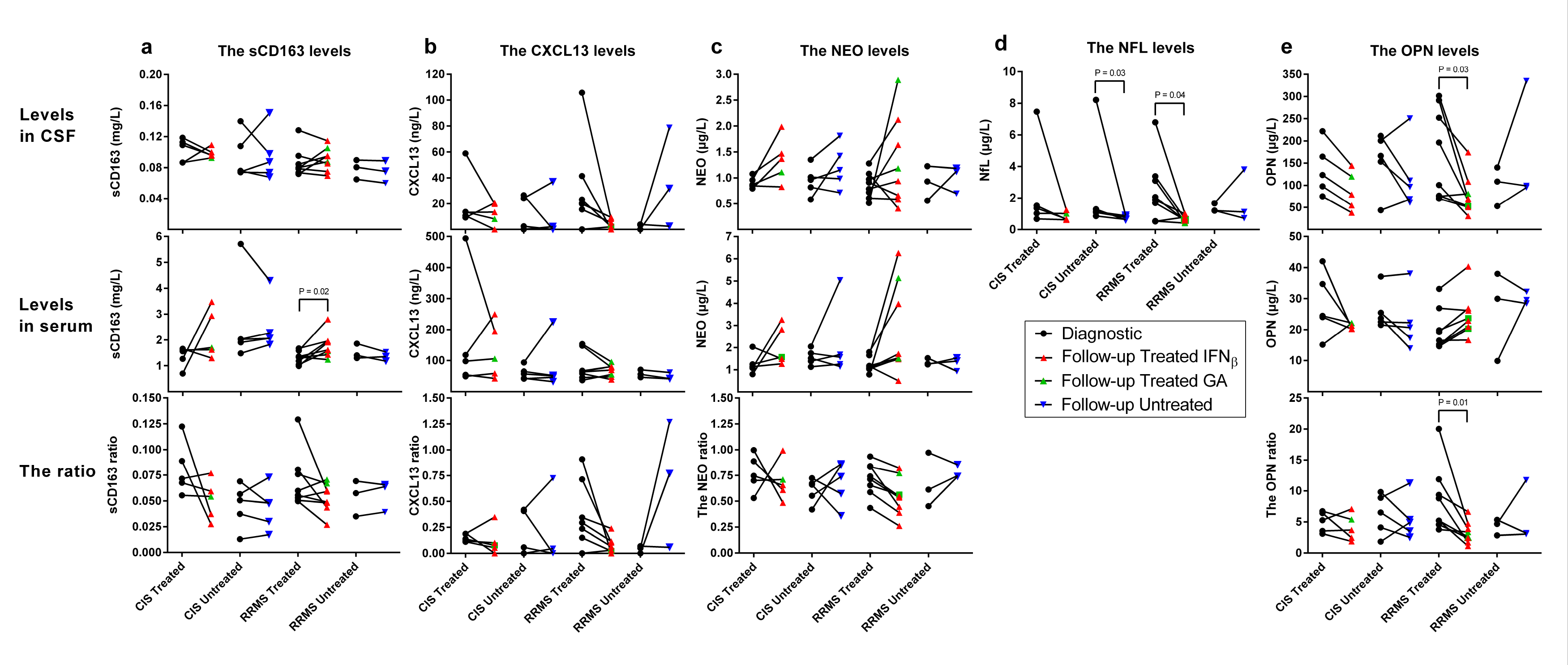


**Figure S1. Biomarker levels in either treated or untreated patients with RRMS or CIS.** Changes in the CSF and serum levels in the follow-up cohort in treated and untreated patients are shown in figure S1a to S1e. In figure S1a we present the levels of CSF sCD163, serum sCD163, and ratio sCD163 for treated and untreated patients with CIS or RRMS at the time of diagnosis and after a minimum of 1 year follow-up time. Figures (S1b-S1e) are likewise produced for CXCL13, NEO, NfL, and OPN. Each column shows the biomarker levels in treated or untreated patients with CIS or RRMS. A U-test (Mann-whitney non-parametric test) of difference between diagnostic and follow-up levels and a significant difference p<0.05 is marked by a bar and corresponding p-values. Patients were treated with either glatiramer acetate (n=3, green triangle) or interferon beta (n=10, red triangle), or untreated (n=8, blue triangle). Abbreviations: CIS (clinically isolated syndrome), RRMS (relapsing remitting MS), NEO (neopterin), NfL (neurofilament light polypeptide), OPN (osteopontin), GA (glatiramer acetate), IFN-β (interferon beta).

| Table S23. Biomarker levels in either treated or untreated patients with RRMS or CIS. | | | | | | | | | | | | |
| --- | --- | --- | --- | --- | --- | --- | --- | --- | --- | --- | --- | --- |
| **Characteristics** | **MDL CIS-T** | **MFL CIS-T** | **U-test** | **MDL CIS-UT** | **MFL CIS-UT** | **U-test** | **MDL RRMS-T** | **MFL RRMS-T** | **U-test** | **MDL RRMS -T** | **MFL RRMS-UT** | **U-test** |
| **CSF sCD163 (mg/L)** | **0.109** | **0.096** | **0.802** | **0.076** | **0.087** | **0.802** | **0.079** | **0.091** | **0.375** | **0.081** | **0.075** | **0.700** |
| **range** | **(0.086 - 0.119)** | **(0.093 - 0.109)** |  | **(0.074 - 0.140)** | **(0.070 - 0.150)** |  | **(0.072 - 0.128)** | **(0.070 - 0.115)** |  | **(0.065 - 0.090)** | **(0.060 - 0.089)** |  |
| **Serum sCD163 (mg/L)** | **1.564** | **1.706** | **0.095** | **2.026** | **2.067** | **0.413** | **1.33** | **1.761** | **0.021*** | **1.404** | **1.353** | **0.700** |
| **range** | **(0.703 - 1.657)** | **(1.298 - 3.476)** |  | **(1.481 - 5.712)** | **(1.821 - 4.291)** |  | **(0.992 - 1.673)** | **(1.238 - 2.804)** |  | **(1.298 - 1.859)** | **(1.179 - 1.530)** |  |
| **sCD163 ratio** | **0.072** | **0.054** | **0.095** | **0.051** | **0.047** | **0.802** | **0.058** | **0.054** | **0.193** | **0.057** | **0.064** | **0.900** |
| **range** | **(0.055 - 0.122)** | **(0.027 - 0.077)** |  | **(0.013 - 0.069)** | **(0.017 - 0.073)** |  | **(0.050 - 0.129)** | **(0.027 - 0.071)** |  | **(0.035 - 0.069)** | **(0.039 - 0.066)** |  |
| **CSF CXCL13 (ng/L)** | **13.28** | **13.49** | **0.802** | **2.478** | **0.001** | **0.683** | **20.11** | **3.703** | **0.058** | **3.580** | **31.72** | **0.400** |
| **range** | **(9.277 - 58.97)** | **(0.001 - 20.62)** |  | **(0.001 - 26.55)** | **(0.001 - 36.91)** |  | **(0.001 - 105.8)** | **(0.001 - 9.319)** |  | **(0.001 - 3.895)** | **(2.456 - 78.78)** |  |
| **Serum CXCL13 (ng/L)** | **98.16** | **106.1** | **0.944** | **57.44** | **50.87** | **0.802** | **64.74** | **73.80** | **0.777** | **56.19** | **42.28** | **0.400** |
| **range** | **(49.33 - 493.9)** | **(42.57 - 249.6)** |  | **(41.79 - 93.92)** | **(32.30 - 224.9)** |  | **(36.91 - 153.6)** | **(38.81 - 95.44)** |  | **(46.66 - 70.67)** | **(41.25 - 62.08)** |  |
| **CXCL13 ratio** | **0.140** | **0.077** | **0.151** | **0.058** | **<0.000** | **0.667** | **0.266** | **0.048** | **0.104** | **0.051** | **0.769** | **0.200** |
| **Range** | **(0.112 - 0.195)** | **(<0.00 - 0.349)** |  | **(<0.00 - 0.421)** | **(<0.00 - 0.726)** |  | **(<0.00 - 0.909)** | **(<0.00 - 1.239)** |  | **(<0.00 - 0.069)** | **(0.058 - 1.269)** |  |
| **CSF NEO (µg/L)** | **0.869** | **1.368** | **0.095** | **0.964** | **1.152** | **0.310** | **0.849** | **1.061** | **0.495** | **0.932** | **1.130** | **0.900** |
| **range** | **(0.793 - 1.080)** | **(0.827 - 1.987)** |  | **(0.580 - 1.351)** | **(0.713 - 1.813)** |  | **(0.514 - 1.279)** | **(0.408- 2.891)** |  | **(0.558 - 1.226)** | **(0.930 - 1.525)** |  |
| **Serum NEO (µg/L)** | **1.132** | **1.565** | **0.056** | **1.513** | **1.559** | **0.944** | **1.120** | **1.622** | **0.083** | **1.261** | **1.387** | **0.900** |
| **range** | **(0.796 - 2.034)** | **(1.261 - 3.260)** |  | **(1.130 - 2.043)** | **(1.142 - 5.040)** |  | **(0.786 - 1.793)** | **(0.496 - 6.257)** |  | **(1.231 - 1.520)** | **(1.387 - 1.525)** |  |
| **NEO ratio** | **0.747** | **0.656** | **0.413** | **0.661** | **0.739** | **0.413** | **0.727** | **0.541** | **0.065** | **0.613** | **0.748** | **0.700** |
| **range** | **(0.531 - 0.996)** | **(0.486 - 0.993)** |  | **(0.420 - 0.724)** | **(0.360 - 0.861)** |  | **(0.435 - 0.935)** | **(0.262 - 0.822)** |  | **(0.453 - 0.972)** | **(0.741 - 0.854)** |  |
| **CSF NfL (µg/L)** | **1.361** | **0.672** | **0.056** | **1.140** | **0.781** | **0.032*** | **1.976** | **0.657** | **0.038*** | **1.212** | **1.120** | **0.700** |
| **range** | **(0.682 - 7.472)** | **(0.620 - 1.242)** |  | **(0.866 - 8.209)** | **(0613. - 0.896)** |  | **(0.524- 6.798)** | **(0.415 - 1.017)** |  | **(1.201 - 1.671)** | **(0.723 - 3.813)** |  |
| **CSF OPN (µg/L)** | **123.2** | **78.94** | **0.222** | **166.5** | **96.20** | **0.532** | **148.4** | **66.93** | **0.028*** | **108.4** | **98.65** | **0.900** |
| **range** | **(74.83- 221.8)** | **(38.12- 144.2)** |  | **(43.66 -211.2 )** | **(60.73 - 250.7)** |  | **(69.81 - 301.6)** | **(30.31 - 174.3)** |  | **(53.0 - 140.2)** | **(94.82 - 335.0)** |  |
| **Serum OPN (µg/L)** | **24.48** | **21.30** | **0.151** | **23.70** | **20.71** | **0.222** | **17.88** | **23.32** | **0.104** | **29.96** | **29.56** | **0.900** |
| **range** | **(15.14 - 42.04)** | **(20.23 - 22.19)** |  | **(21.50 - 37.16)** | **(13.95 - 38.09)** |  | **(14.64 - 33.13)** | **(16.63 - 40.37)** |  | **(9.901 - 38.00)** | **(28.47 - 32.22)** |  |
| **OPN ratio** | **5.276** | **3.707** | **0.667** | **6.539** | **4.900** | **0.802** | **7.006** | **2.999** | **0.007*** | **4.681** | **3.207** | **0.900** |
| **range** | **(3.110 - 6.730)** | **(1.884 - 7.088)** |  | **(1.843 - 9.821)** | **(2.526 - 11.26)** |  | **(3.771 - 20.03)** | **(1.126 - 6.660)** |  | **(2.853 - 5.353)** | **(3.062 - 11.77)** |  |

**Table S23.** **Biomarker levels in either treated or untreated patients with RRMS or CIS.** Changes in the CSF and serum levels in the follow-up cohort in treated and untreated patients are shown in table 22S (below). A U-test (Mann-Whitney non-parametric test of difference between diagnostic and follow-up levels and a significant difference p<0.05 is marked by *. Abbreviations: CIS (clinically isolated syndrome), RRMS (relapsing remitting MS), NEO (neopterin), NfL (neurofilament light polypeptide), OPN (osteopontin), MDL (median diagnostic level), MFL (median follow-up level), CIS-T (CIS-treated), UT (CIS-untreated).

Table S24: Biomarker SD (standard deviation), and percentiles****

**Table S24: Biomarker SD (standard deviation), and percentiles.** For each group the standard deviation (SD), 5 percentile, 50 percentile, and 95 percentile have been calculated for each biomarker. The Symptomatic Control (SC) group 95 percentile marked by a red box is used for biomarker cut-points.

**Contact**

For further information please contact [mortenleifms@gmail.com](mailto:mortenleifms@gmail.com)
